# Supplementary material for: Temporal organization of stride-to-stride variations contradicts predictive models for sensorimotor control of footfalls during walking
Source: PLoS One. 2023 Aug 24;18(8):e0290324. doi: 10.1371/journal.pone.0290324 (PMC10449478; doi:10.1371/journal.pone.0290324)
Supplement: Attachment — Submitted filename: RR_Mangalam_et_al__2023a_PLoS_One.pdf [file pone.0290324.s002.pdf]

Authors' Response to Reviews of

Temporal organization of stride-to-stride variations contradicts predictive models for sensorimotor control of footfalls during walking

M. Mangalam

PLoS One, PONE-D-23-13654

RC: ***Reviewers' Comment***, AR: Authors' Response, ☐ Manuscript Text

1. Reviewer #1

RC: ***This study analyzed gait cycle variability during self-paced and visual-cue-paced walking. Variability of gait cycle and associated time series were characterized for (i) visual cues (visual metronome), (ii) footfalls (gait cycle), and (iii) asynchronies between visual cues and footfalls by using several metrics, including: (1) ergodicity breaking based on the Thirumalai-Mountain metric, (2) Hurst exponent based on the standard DFA, (3) singularity spectrum (multi-fractal spectrum) based on Chhabra and Jensen's method. Results of the analysis were novel and interesting. Thus, the manuscript deserves publication. However, I have major concerns on the interpretation of the results. Indeed, the interpretation of the authors is well summarized in the title of the manuscript: "Temporal organization of stride-to-stride variations contradicts predictive models for sensorimotor control of walking." To be brief, I don't think the predictive models of human gait has not been gained popularity (although there might be many such models in the field of robotics for bipedal robot locomotion), unlike those for motor control of upper limb voluntary movements. Thus, the term "contradiction" to non-validated predictive models sounds slightly odd, at least for me. Although I am hopeful that I would be convinced by the rebuttal arguments, I recommend to lower the tone of statement in the revised manuscript.***

AR: Thank you for these kind words. We have duly addressed all concerns as detailed below.

RC: ***As mentioned above, the gait and posture are classified as the automatic movement, and they are substantially different from voluntary movements. On the other hand, the visual metronome conditions used in this study provide a continuous guide for the timing of forthcoming footfall, which might make such a gait more or less voluntary, compared to the natural SPW. The current study (particularly the interpretation of the results) is mixed up those two different kinds of motor control, and derived a conclusion in a unified manner about the sensorimotor control of gait in general. Since the major outcomes on the ergodicity breaking (Fig. 4 bottom), the scale-free like behaviors in DFA (Fig. 6 bottom), and cascade-like intermittency in singularity spectrum (Fig. 8 bottom) are all for the asynchrony between visual cues and footfalls, and the asynchrony is heavily dependent on the voluntary decision making on the foot placement, the assertion of the contradiction to the predictive control should be limited to such a gait pattern with the voluntary attempt to regulate the footfall timings, not necessarily to the gait control, including the natural SPW in general. I think the title of this paper should be weaken in this way. For example, "Temporal organization of stride-to-stride variations contradicts predictive models for sensorimotor control during metronomed (or volitional) walking."***

AR: Thank you for highlighting these issues. We have modified the title of the manuscript to address these issues:

> Temporal organization of stride-to-stride variations contradicts predictive models for sensorimotor control of footfalls during walking

We have deliberately chosen not to confine the title solely to pacing since footfalls in the control condition demonstrated identical fractal, multifractal, and ergodic properties as footfalls in the paced conditions.

RC: ***The other concern is also related to the interpretation of the authors about the predictive model. Particularly, rationale to derive their conclusion from the ergodicity breaking. It seems that the authors consider the predictive control and the ergodicity of the gait cycle variability equivalent. Can it be justified theoretically? In my opinion, simple interventions to the gait rhythm, which are determined (generated) based on the state of the gait control system only for the present (i.e., only for the past one cycle) might be able to generate long-range correlation in the gait cycle variability. Such simple but nonlinear (and/or impulsive) interventions, for example, might include the phase resetting and the intermittent control that utilizes a stable manifold of unstable limit cycle of the gait system with no active feedback control. See Fu et al (Biological Cybernetics 114 (1), 95-111, 2020), for example. Note that the intermittent control in this case is not activated by a threshold-crossing that leads to the risk of fall, but the active feedback controller is switched off when the state of the system visits a neighbor of the stable manifold, by which the state point of the system might exhibit a slow sliding motion along the stable manifold in a manner of stride-to-stride basis (a transiently converging sequence of the state points in terms of Poincare mapping that observes the state point at every switching-off event). Note that my point is not the details of the nonlinear feedback control, but the gait fractality (and possibly ergodicity breaking phenomena) could be emerged through some control mechanisms (such as the phase resetting and the intermittent controller, either predictive or non-predictive) that modulate the gait rhythm based on the state of the gait control system only for the present cycle. If so, ergodicity breaking does not necessarily conflict with the models about the sensorimotor control that correct the timings of the footfall from one stride to the next. This is why I would not be able to agree with the interpretation of the authors.***

AR: We have addressed all these concerns by adding the following section in the introduction:

> **Ergodicity as a foundation for linearization-based models of gait control, with or without prediction**

RC: ***There are some studies (e.g., Fluctuation of stride time intervals during walking with smartphone, S Yano, L Dimalanta, Y Suzuki, T Nomura, 2019 IEEE 1st Global Conference on Life Sciences and Technologies, DOI:10.1109/LifeTech.2019.8884072) showing that the cognitive load decreases the persistency in gait cycle variability. From Fig. 6 (middle), I can see a similar tendency for SPPS, GRPS and URPS. However, since the positive persistency is lost also in the visual metronome, the decrease in the persistency for the gait cycle variability could be caused by the metronome. Please discuss possible causes of the loss of the fractality in the gait cycle variability.***

AR: We have discussed this possibility in the Discussion section (see main text for references):

> Furthermore, increased cognitive could have contributed to reducing fractal profile in Yano et al. [**?**]. However, the strong multifractal evidence of nonlinearity and of ergodicity breaking limit the usefulness or generality of inventories of independent components or constraints whose effects may be genuine but fleeting or challenging to reproduce. Despite previous attempts to construe the $1/f$ form of strides as a linear process (e.g., [**?**]) submitting to the independent effects of independent constraints such

as cognitive load, the present work confirms both that gait is more than just $1/f$ and more than just linear. The origins of the multifractal cascade are evident in the ergodicity-breaking dynamics of strides and asynchronies suggest espousing more stochastic notions of causation that do not always allow identification of independent effects [?].

RC: ***Please elaborate more about how the horizontal bar exhibited as the visual cues moved. Does the bar move with a constant velocity for each desired gait cycle? That is, for example, let me consider two stride intervals T1 and T2. If T1>T2, and if the bar moves at a constant velocity, it should move faster for T2 than for T1. In this case, the velocity of the bar is very informative for the subject, by which one can predict next instant of the footfall. Is this ok? Did you provide such information to the subject on purpose?***

AR: We have added these details in the Methods section:

> The bar's velocity was derived from the average velocity of each participant, as determined during the self-paced walking trial. Subsequently, fluctuations were introduced, superimposing upon this mean velocity, and their characteristics were based on the selected distribution (e.g., pink, white, etc.). That being said, the bar's velocity could be informative for the participants, allowing them to predict the next instant of the footfall. For instance, let us consider two stride intervals, T1 and T2. If T1 is greater than T2, and the bar consistently moves at this constant velocity, it will cover a greater distance in T2 compared to T1. The participants were instructed to match their right footfalls to the instant the moving bar reached the stationary top bar and to match their left footfalls to the instant the moving bar reached the stationary bottom bar.

RC: ***Was the subject forced to change the direction (maybe slightly) along the track field? Did the subject walk straight? I am asking this because the walk way shown in Fig. 2 is curved.***

AR: We have added the following note in the Methods section:

> The participant was not forced to change along their direction along the track. The curved nature of the track naturally led the participants to change direction during their walk.

RC: ***Fig. 2: Please do not call the stride between two footprints as "stride interval" since the stride interval is reserved for the stride time interval.***

AR: We have corrected this typo.

RC: ***l292: (ii) should be (iii)***

AR: We have corrected this typo.

RC: ***Fig. 3 top traces: Why did the graphs of cues appear with the artifact of digitization? The resolution of the graphs should be much higher. Was such low resolution visual metronome data used in the experiment?***

AR: Thank you for bringing this to our attention. The digitization effect observed in the top traces of Fig 3 is a result of the Gaussian distribution and the low resolution of the metronome signal.

RC: ***Fig. 7: I don't think the illustration of multi-fractal (distributed singularity along time axis) is correct rigorously speaking, although it might be intuitively helpful for some people who are not familiar with the concept. In reality, different strength of singularity for multiple scales are distributed in a nested manner. I recommend to remove this part of Fig. 7, or make it more mathematically sound.***

AR: Thanks for pointing this issue. However, we insist that our portrayal of the multifractal spectrum as an extension of fractal statistics is justified for several reasons:

- We base our approach on Ihlen's (2012) simulated demonstration, which shows that the time sequence accurately portrays the singular spectrum, effectively describing each singularity's frequency. This demonstrates the reliability and validity of our method in capturing the multifractal nature of the data.
- Turiel et al.'s (2007) microcanonical approach is also considered in our analysis, which estimates singularities for each point in a measurement series. By incorporating this approach, we account for the intricate variations in the data, further enhancing the accuracy of our multifractal representation.
- While we acknowledge using the canonical approach to estimate singularities by bin, we recognize that it might not capture the full complexity of the underlying multifractal structure. Despite this limitation, we have taken this approach as it still provides valuable insights into the multifractal nature of the data, allowing us to make meaningful interpretations.
- Furthermore, we acknowledge the possibility of a more accurate portrayal of singularities through a nested arrangement. While this approach might yield more precise results, it can also be more computationally intensive and challenging to implement. Therefore, considering the trade-offs between accuracy and feasibility, we have chosen a representation that balances interpretability and computational efficiency.

In conclusion, our portrayal of the multifractal spectrum as an extension of fractal statistics is well-founded, drawing on validated methodologies and considering different approaches to estimating singularities. While there might be room for improvement with a nested arrangement, our current approach provides valuable insights into the multifractal characteristics of the data, contributing to a better understanding of its underlying complexity.

Ihlen, E. A. (2012). Introduction to multifractal detrended fluctuation analysis in Matlab. *Frontiers in Physiology*, *3*, 141. `https://doi.org/10.3389/fphys.2012.00141`

Turiel, A., Yahia, H., & Pérez-Vicente, C. J. (2007). Microcanonical multifractal formalism—A geometrical approach to multifractal systems: Part I. Singularity analysis. *Journal of Physics A: Mathematical and Theoretical*, *41*(1), 015501. `https://doi.org/10.1088/1751-8113/41/1/015501`

RC: ***Please provide a clear definition of $\delta^2(x(t))$ both in the main text and in the Method section.***

AR: We have defined $\delta^2(x(t))$ in both Results and Methods sections.

> This E_B statistic subtracts the squared total-sample variance, $\langle\overline{\delta^2(x(t))}\rangle^2$, from the average squared subsample variance, $\langle[\overline{\delta^2(x(t))}]^2\rangle$, and divides the resultant by the squared total-sample variance, $\langle\overline{\delta^2(x(t))}\rangle^2$: $\mathrm{EB}(x(t)) = \frac{\langle[\overline{\delta^2(x(t))}]^2\rangle - \langle\overline{\delta^2(x(t))}\rangle^2}{\langle\overline{\delta^2(x(t))}\rangle^2}$; where $\delta^2(x(t))$ is sample-to-sample variance and this relationship is effectively the variance of the sample variance divided by the squared total-sample variance.
